# De Ritis ratio in elderly glioblastoma patients treated with chemoradiation: A comprehensive analysis of serum biomarkers

**DOI:** 10.1093/noajnl/vdad173

**Published:** 2023-12-28

**Authors:** Jina Kim, Hye In Lee, In Ah Kim, Joo Ho Lee, Jaeho Cho, Chan Woo Wee, Hong In Yoon

**Affiliations:** Department of Radiation Oncology, Yonsei Cancer Center, Heavy Ion Therapy Research Institute, Yonsei University College of Medicine, Seoul, Korea; Department of Radiation Oncology, Seoul National University Hospital, Seoul National University College of Medicine, Seoul, Korea; Department of Radiation Oncology, Asan Medical Center, Seoul, Korea; Department of Radiation Oncology, Seoul National University Bundang Hospital, Seoul National University College of Medicine, Seongnam, Korea; Department of Radiation Oncology, Seoul National University Hospital, Seoul National University College of Medicine, Seoul, Korea; Department of Radiation Oncology, Yonsei Cancer Center, Heavy Ion Therapy Research Institute, Yonsei University College of Medicine, Seoul, Korea; Department of Radiation Oncology, Yonsei Cancer Center, Heavy Ion Therapy Research Institute, Yonsei University College of Medicine, Seoul, Korea; Department of Radiation Oncology, SMG-SNU Boramae Medical Center, Seoul National University College of Medicine, Seoul, Korea; Department of Radiation Oncology, Yonsei Cancer Center, Heavy Ion Therapy Research Institute, Yonsei University College of Medicine, Seoul, Korea

**Keywords:** chemoradiation, De Ritis ratio, elderly, glioblastoma, glucose

## Abstract

**Background:**

We aimed to comprehensively investigate the prognostic value of pretreatment laboratory parameters in elderly patients with glioblastoma treated with temozolomide (TMZ)-based chemoradiation.

**Methods:**

Patients aged ≥ 65 years from 4 institutions with newly diagnosed IDH-wild-type glioblastoma who received radiotherapy (RT) with concurrent TMZ between 2006 and 2021 were included. Patient factors (age, Karnofsky performance status (KPS), temporalis muscle thickness), molecular factors (MGMT promoter methylation, EGFR amplification, TERT promoter mutation, and TP53 mutation status), treatment factors (extent of resection, and RT dose), and pretreatment laboratory parameters (serum De Ritis ratio, glucose level, neutrophil-to-lymphocyte ratio, platelet count, and systemic immune-inflammation index) were included in the analysis. The primary endpoint was overall survival (OS).

**Results:**

In total, 490 patients were included in the analysis. The median follow-up period was 12.3 months (range, 1.6–149.9 months). Median OS was significantly prolonged in patients with De Ritis ratio < 1.2 (18.2 vs 15.3 months, *P* = .022) and in patients with glucose level < 150 mg/dL (18.7 vs 16.5 months, *P* = .034) per univariate analysis. In multivariate analysis, KPS ≥ 70, MGMT promoter methylation, extent of resection greater than partial resection, De Ritis ratio < 1.2, and glucose level < 150 mg/dL were significant prognostic factors for improved OS.

**Conclusions:**

Along with well-known prognostic factors, pre-RT serum biomarkers, including the De Ritis ratio and glucose level, also had prognostic value in elderly patients with glioblastoma treated with TMZ-based chemoradiation.

**Key Points**

- Prognostic value of routine lab parameters in elderly GBM patients was studied.
- De Ritis ratio < 1.2 and glucose level < 150 mg/dL were linked to prolonged OS.
- De Ritis ratio and glucose level were also significantly associated with OS in MVA.

**Importance of the Study**

In this study, we performed a comprehensive analysis of various routinely obtained laboratory parameters together with patient-, molecular-, and treatment-related factors in elderly patients with glioblastoma treated with temozolomide-based chemoradiation. Interestingly, we found out that De Ritis ratio < 1.2 and glucose level < 150 mg/dL were linked to prolonged overall survival. To the best of our knowledge, this is the first study to reveal an association between the De Ritis ratio and survival outcomes in elderly patients with glioblastoma.

Glioblastoma (GBM) is the most common malignant primary brain tumor in adults, and its prognosis still remains dismal.^1^ The standard treatment option for GBM involves surgical removal of the tumor, followed by administration of temozolomide (TMZ)-based chemoradiation.^2^ The well-known prognostic factors for glioblastoma include age, performance status, surgical extent, and O^6^-methylguanine-DNA methyltransferase (MGMT) promoter methylation status.^3–5^ The median survival of elderly patients is significantly shorter than that of younger patients, partly because of unfavorable tumor biology, poor performance status, comorbidities, and vulnerability to treatment-related toxicities.^6^

Routine laboratory parameters are easily available at a relatively low cost and convey abundant information about patients’ conditions.^7^ Inflammatory indices obtained from routine blood tests are associated with the prognosis of patients with various types of solid tumors. An elevated neutrophil-to-lymphocyte ratio (NLR) is associated with a poor prognosis in lung, breast, colorectal, and gastric cancers.^8–11^ The systemic immune-inflammation index (SII), which is easily calculated using neutrophil, platelet, and lymphocyte counts, has also been shown to predict survival outcomes in numerous cancer types.^12^ The prognostic value of the De Ritis ratio (aspartate transaminase [AST]/alanine transaminase [ALT]) was discovered in atypical meningiomas, urothelial cancers, and renal cell carcinomas, and this may be partly explained by the fact that AST is linked with high tumor cell proliferation rate.^13–16^ Glucose levels are also associated with the prognosis of breast, ovarian, nonsmall cell lung cancers, and glioblastoma.^17–20^ However, limited data exist on the prognostic value of routine laboratory parameters in patients with GBM.

In this study, we aimed to investigate the prognostic value of pretreatment laboratory parameters routinely obtained from elderly patients with GBM treated with TMZ-based chemoradiation. We performed a comprehensive analysis of various laboratory parameters after adjusting for patient-, molecular-, and treatment-related factors.

## Materials and Methods

### Study Population

The medical data of a multi-institutional elderly GBM cohort from 4 Korean institutions between January 2006 and December 2021 were reviewed for this study. The patient inclusion criteria were as follows: (a) age ≥ 65 years at the time of diagnosis, (b) newly diagnosed isocitrate dehydrogenase (IDH)-wild-type GBM, and (c) underwent biopsy or surgical resection followed by TMZ-based chemoradiation. Patients diagnosed with double primary malignancies were excluded. This study was approved by the Institutional Review Board of each participating institution (Yonsei University Health System, Severance Hospital IRB approval no. 4-2022-0126), and the requirement for informed consent was waived because of the retrospective nature of this study.

### Treatment and Response Evaluation

The extent of resection was defined based on immediate postoperative magnetic resonance imaging (MRI) taken within 48–72 h after surgery in all patients and was classified as follows: (1) gross total resection: no or less than 1% evidence of preoperative tumor volume, (2) near total resection: residual tumor between 1% and 5%, (3) subtotal resection: residual tumor between 5% and 20%, (4) partial resection: residual tumor between 20% and 50%, and (5) biopsy: residual tumor greater than 50%. All patients underwent profiling to determine the MGMT promoter methylation status using methylation-specific polymerase chain reaction. In a subset of patients, mutations in the telomerase reverse transcriptase (TERT) promoter and tumor protein p53 (TP53) were examined using next-generation sequencing.

Laboratory results, including serum De Ritis ratio, glucose level, neutrophil count, lymphocyte count, and platelet count obtained within 1–7 days prior to the initiation of radiotherapy (RT), were collected. The SII was calculated by multiplying the neutrophil count by the platelet count and dividing the resulting value by the lymphocyte count. Temporalis muscle thickness (TMT) measurements were conducted on preoperative contrast-enhanced T1-weighted MRI images by determining the length of an imaginary line perpendicular to the long axis of the temporal muscle at the orbital roof level. Patients with mean TMT values ≤ 6.3 mm for men and ≤5.2 mm for women were classified as narrow, while those with mean TMT values exceeding these thresholds were classified as having a normal TMT.^21^

All patients received RT in combination with concurrent and adjuvant TMZ as per the Stupp protocol (75 mg/m^2^ of body surface area per day, 7 days a week, from the first to the last day of RT), followed by 6 cycles of adjuvant TMZ (150–200 mg/m^2^ for 5 days in each 28-day cycle). Patients were treated with either 3-dimensional conformal RT (3D-CRT) or intensity-modulated RT (IMRT). RT was administered in either the conventional or hypofractionated dose scheme based on the physicians’ discretion.

For tumor response assessment after completion of RT, follow-up evaluations with neurological assessment and brain MRI were performed every 3 months for up to 2 years and every 4–6 months thereafter and were judged based on the response assessment in the Neuro-Oncology criteria.

### Statistical Analysis

The cutoff values used for De Ritis ratio and serum glucose level analyses in this study were 1.2 and 150 mg/dL, respectively. The primary endpoint was overall survival (OS). OS was defined as the time from the date of the initial surgery to death from any cause. The secondary endpoint was progression-free survival (PFS), defined as disease progression or death, whichever occurred first. The Kaplan–Meier method with the log-rank test was used for OS and PFS analyses. For univariate and multivariate analyses, Cox regression with the enter method was used. Statistical significance was set at *P* < .05, and IBM SPSS Statistics for Windows version 25.0 (IBM Corp., Armonk, NY, USA) was used for the statistical analyses.

## Results

A total of 490 patients were included in this analysis. The patient and tumor characteristics are presented in Table 1. Of the 490 patients, TERT promoter and TP53 gene mutation status data were available for 217 (44.3%). 3D-CRT was administered to 158 (32.2%) patients and IMRT to 332 (67.8%) patients. Approximately two-thirds of the patients (*N* = 324, 66.1%) received conventionally fractionated RT, while one-third (*N* = 166, 33.9%) received hypofractionated RT. Median RT dose in biological equivalence dose was 72 Gy (range, 49.1–87.5 Gy, *α*/*β* = 10), which corresponds to 60 Gy in 30 fractions.

**Table 1.** Patient and Tumor Characteristics.

		No/median	%/Range
Age (years)		69	65–86
Karnofsky performance status	<70	200	40.8
	≥70	290	59.2
EGFR status	Not amplified	148	30.2
	Amplified	69	14.1
	Unknown	273	55.7
TERT status	Wild-type	100	20.4
	Mutated	117	23.9
	Unknown	273	55.7
TP53 status	Wild-type	141	28.8
	Mutated	76	15.5
	Unknown	273	55.7
MGMT status	Unmethylated	267	54.5
	Methylated	223	45.5
Temporal muscle thickness	Normal	55	11.2
	Narrow	435	88.8
Resection extent	PR, Biopsy	116	23.7
	GTR, NTR, STR	374	76.3
Radiotherapy dose (BED, Gy)		72	49.1–87.5
Neutrophil-to-lymphocyte ratio		2.99	0.19–35.42
Platelet count (×1000 cells/mL)		229	79–507
SII (×1000 cells/μL)		728.4	34.8–7326.3
De Ritis ratio		0.94	0.08–4.14
Glucose level (mg/dL)		112	26–539

Abbreviations: No, number; EGFR, epidermal growth factor receptor; TERT, telomerase reverse transcriptase; TP53, tumor protein 53; MGMT, O^6^-methylguanine-DNA-methyltransferase; PR, partial resection; GTR, gross total resection; NTR, near total resection; STR, subtotal resection; BED, biological effective dose; SII, systemic immune-inflammation index.

**Figure 1.**
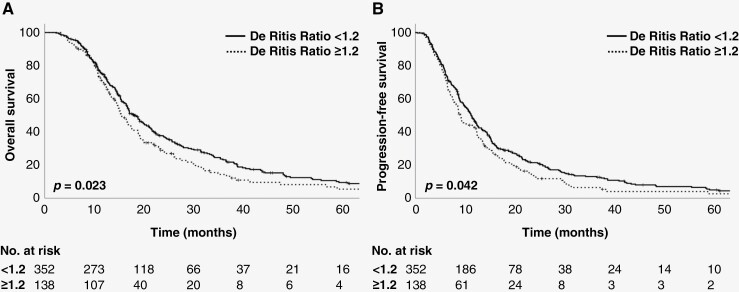
Kaplan–Meier estimates of (A) overall survival and (B) progression-free survival according to De Ritis ratio.

**Figure 2.**
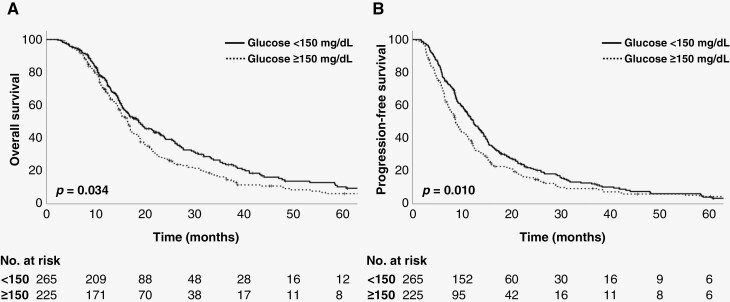
Kaplan–Meier estimates of (A) overall survival and (B) progression-free survival according to glucose level.

With a median follow-up of 17.5 months for survivors (range, 3.3–149.9 months), median OS was 17.0 months for the entire cohort. OS of patients with De Ritis ratio < 1.2 was significantly higher than that of patients with De Ritis ratio ≥ 1.2 (median OS 18.2 vs 15.3 months, *P* = .022) (Figure 1A). PFS was also significantly higher in patients with De Ritis ratio < 1.2 compared to their counterparts (median PFS 11.2 vs 9.1 months, *P* = .042) (Figure 1B). Similar results were observed for the serum glucose levels. Median OS of patients with serum glucose level < 150 mg/dL was significantly longer than that of patients with serum glucose level ≥ 150 mg/dL (median OS 18.7 vs 16.5 months, *P* = .034) (Figure 2A). Median PFS was also significantly longer in patients with serum glucose level < 150 mg/dL than that of patients with serum glucose level ≥ 150 mg/dL (median PFS 12.3 vs 8.7 months, *P* = .010) (Figure 2B).

Results of subgroup analysis with combined phenotypes of De Ritis ratio and MGMT are shown in Supplementary Figure 1. Patients with both low De Ritis ratio and methylated MGMT promoter had the best prognosis, followed by those with high De Ritis ratio and methylated MGMT promoter (median OS 25.0 vs 14.8 months, *P* < .001). Those with both high De Ritis ratio and unmethylated MGMT promoter had the worst prognosis (median OS 13.1 months).

The results of univariate and multivariate analyses for OS are shown in Table 2. A higher De Ritis ratio (≥1.2) and higher glucose levels (≥150 mg/dL) were significant prognostic factors for poor OS in univariate analysis. Both variables remained significant in the multivariate analysis. In addition, the De Ritis ratio and serum glucose level were identified as a significant prognostic factor for OS in the subgroup of patients with available TERT promoter, TP53, and EGFR status (*N* = 217) (Table 3).

**Table 2.** Univariate and Multivariate Analysis for Overall Survival in All Patients (*N* = 490).

	Univariate analysis			Multivariate analysis		
	HR	95% CI	P value	HR	95% CI	P value
Age (≥70 years vs <70 years)	1.33	1.08–1.63	.007	N.S.		
KPS (≥70 vs <70)	0.53	0.43–0.65	<.001	0.53	0.43–0.66	<.001
MGMT (methylated vs unmethylated)	0.57	0.46–0.70	<.001	0.53	0.43–0.66	<.001
Temporal muscle thickness (normal vs narrow)	0.59	0.44–0.80	.001	N.S.		
Resection extent (GTR, NTR, STR vs PR, biopsy)	0.66	0.53–0.84	.001	0.75	0.59–0.96	.021
Neutrophil-to-lymphocyte ratio (<4.0 vs ≥4.0)	0.97	0.78–1.20	.743	N.S.		
Platelet count (≥230 vs <230 K/μL)	0.91	0.74–1.11	.351	N.S.		
SII (<730 × 1000 vs ≥730 × 1000 cells/μL)	0.91	0.74–1.12	.374	N.S.		
De Ritis ratio (≥1.2 vs <1.2)	1.29	1.04–1.61	.023	1.27	1.01–1.59	.038
Glucose level (≥150 vs <150 mg/dL)	1.25	1.02–1.53	.034	1.27	1.03–1.57	.025

Abbreviations: HR, hazard ratio; CI, confidence interval; N.S., not significant; KPS, Karnofsky performance status; MGMT, O^6^-methylguanine-DNA-methyltransferase; GTR, gross total resection; NTR, near total resection; STR, subtotal resection; PR, partial resection; SII, systemic immune-inflammation index.

**Table 3.** Univariate and Multivariate Analysis for Overall Survival in Patients with Molecular Factors Available (*N* = 217).

	Univariate analysis			Multivariate analysis		
	HR	95% CI	P value	HR	95% CI	P value
Age (≥70 years vs <70 years)	1.15	0.82–1.63	.419	N.S.		
KPS (≥70 vs <70)	0.47	0.34–0.67	<.001	0.42	0.29–0.60	<.001
MGMT (methylated vs unmethylated)	0.44	0.31–0.62	<.001	0.36	0.25–0.52	<.001
EGFR (amplified vs not amplified)	0.84	0.58–1.23	.373	N.S.		
TERT (mutated vs wild-type)	0.97	0.69–1.37	.868	N.S.		
TP53 (mutated vs wild-type)	1.69	1.19–2.41	.004	N.S.		
Temporal muscle thickness (normal vs narrow)	0.54	0.29–1.00	.051	N.S.		
Resection extent (GTR, NTR, STR vs PR, biopsy)	0.41	0.27–0.62	<.001	0.45	0.30–0.69	<.001
Neutrophil-to-lymphocyte ratio (<4.0 vs ≥4.0)	0.67	0.46–0.98	.041	N.S.		
Platelet count (≥230 vs <230 K/μL)	0.73	0.52–1.03	.071	N.S.		
SII (<730 × 1000 vs ≥730 × 1000 cells/μL)	0.67	0.47–0.94	.022	N.S.		
De Ritis ratio (≥1.2 vs <1.2)	1.59	1.10–2.28	.013	1.49	1.01–2.19	.046
Glucose level (≥150 vs <150 mg/dL)	1.66	1.17–2.34	.004	1.50	1.05–2.15	.027

Abbreviations: HR, hazard ratio; CI, confidence interval; N.S., not significant; KPS, Karnofsky performance status; MGMT, O^6^-methylguanine-DNA-methyltransferase; EGFR, epidermal growth factor receptor; TERT, telomerase reverse transcriptase; TP53, tumor protein 53; GTR, gross total resection; NTR, near total resection; STR, subtotal resection; PR, partial resection; SII, systemic immune-inflammation index.

## Discussion

In this study, we investigated the prognostic value of pretreatment laboratory data in elderly patients with GBM treated with TMZ-based chemoradiotherapy. Well-known prognostic factors in GBM, including lower performance status, unmethylated MGMT, and smaller resection extent, are predictive of OS. Interestingly, De Ritis ratio ≥ 1.2 and serum glucose level ≥ 150 mg/dL were also associated with poor prognosis. The De Ritis ratio and serum glucose level were highly significant factors, as shown in the multivariate analysis. However, no association was observed between the NLR, platelet count, or SII and survival outcomes in our study.

At a cutoff value of 1.2, patients with a lower De Ritis ratio had higher OS and PFS than their counterparts. Although we believe that this is the first study to reveal the prognostic value of the De Ritis ratio in elderly patients with GBM, the prognostic value of the De Ritis ratio in solid tumors is not new. Bezan et al. first observed that an elevated preoperative De Ritis ratio was associated with poor metastasis-free survival and OS in patients with nonmetastatic renal cell carcinoma.^14^ The authors hypothesized that increased tissue damage and high tumor cell turnover affect AST to a larger extent than ALT, which partly explains why an elevated De Ritis ratio is associated with poor survival outcomes. The fact that AST plays a role in the malate-aspartate shuttle pathway, facilitating cytoplasmic–mitochondria transfer of the nicotinamide adenine dinucleotide hydrogen during aerobic glycolysis, may also clarify why increased De Ritis ratio is linked to the increased glucose metabolism observed in cancer cells.^15^ In a meta-analysis including 9,400 patients from 18 studies, high pretreatment De Ritis ratio was associated with poor OS (hazard ratio [HR] 1.70, 95% confidence interval [CI] 1.37–2.09, *P* < .001) and recurrence-free survival (HR 1.51, 95% CI 1.15–1.99, *P* = .003).^22^ According to the meta-analysis, association between elevated De Ritis ratio and inferior survival outcome was proven irrespective of the study population, cancer type, primary treatment, clinical stage, cutoff value, analysis method, and sample size. Subgroup analysis based on cancer type revealed that its significance persisted in all cancer types, including urothelial carcinoma, bladder cancer, renal cell cancer, and liver cancer. A recently published study incorporating data from 515 patients treated for oral and oropharyngeal squamous cell cancer identified a De Ritis ratio > 1.44 as an independent prognostic factor for poor survival in head and neck malignancies (HR 1.55, 95% CI 1.12–2.15, *P* = .008).^23^ These findings imply that a simple De Ritis ratio obtained from routine laboratory data can be clinically meaningful in predicting survival outcomes in elderly patients with GBM. De Ritis ratio not only effectively predicts hepatic function but also exhibits a strong correlation with indicators of compromised renal function (eGFR), cardiac strain (NT-proBNP), and inflammation (CRP), implying its potential utility as a surrogate marker for end-organ damage.^24^ Another explanation for the significance of De Ritis ratio as a prognostic factor might be that high De Ritis ratio (>1.2) is associated with sarcopenia.^25^ While narrow TMT was not a significant prognostic factor for OS in the multivariate analysis of our study, Lee et al. have suggested that TMT may be an indicator for sarcopenia, predicting PFS.^26^

In our study, we observed that elevated serum glucose levels were associated with poor OS and PFS. Further analysis incorporating De Ritis ratio and serum glucose level as a single variable revealed that patients with both elevated De Ritis ratio and serum glucose level had the worst prognosis compared to others, significant in both univariate (HR 1.51, 95% CI 1.14–2.01, *P* = .004) and multivariate analysis (HR 1.51, 95% CI 1.13–2.01, *P* = .005; Supplementary Table 1). Previous studies have reported an association between high serum glucose levels and cancer prognosis. In a study involving 148 nondiabetic breast cancer patients, individuals with elevated random blood glucose levels (≥120 mg/dL) had shorter OS (HR 3.50, 95% CI 1.87–6.54, *P* < .001), even after adjusting for other prognostic factors including tumor stage, tumor grade, ethnicity, and body mass index between the 2 groups.^27^ Another study comprising 342 nonsmall cell lung cancer patients revealed that patients with high fasting glucose level (≥126 mg/dL) had significantly higher all-cause mortality risk compared to the patients with normal fasting glucose level (70–99 mg/dL).^18^ An association between high glucose levels and poor clinical outcomes in patients with GBM have also been previously reported. Derr et al. reported progressively shorter median survival with increasing mean glucose levels in newly diagnosed GBM patients, with a 1.57-fold-higher HR in patients with glucose levels > 137 mg/dL than in those with glucose levels < 94 mg/dL.^28^ Adeberg et al. found that in patients with GBM treated with RT, the presence of persistent mild and excessive hyperglycemia was associated with poorer OS.^29^ The time-weighted mean glucose level, calculated from the glucose measurements over a 10-week period during and after RT, was also found to be independently associated with OS in patients with GBM treated with RT and TMZ.^30^ In another study, 1 or more hyperglycemic episodes almost halved the median OS of patients with GBM undergoing adjuvant chemoradiation.^31^ These findings imply that although the cutoff value of 150 mg/dL used in this study is an optimal cutoff value for serum glucose levels, both persistent and episodic hyperglycemia have negative effects on survival outcomes. Interestingly, patients with coexistent GBM and diabetes demonstrated prolonged PFS following metformin therapy.^32^ The “Warburg effect,” characterized by increased aerobic glycolysis in tumor cells, may explain why elevated serum glucose levels are associated with poor prognosis in cancer patients.^33^ Based on these findings, a ketogenic diet has been carefully attempted in patients with GBM; however, no definite benefits have been observed yet.^34–36^ Nonetheless, these studies underscore the clinical significance of serum glucose levels in patients with GBM.

The main limitation of this study is its retrospective design. As only laboratory data prior to RT initiation were available, questions regarding the potential impact of changes in laboratory values during the treatment course on prognosis remain unanswered. Furthermore, the glucose measurements obtained in our study were randomly taken rather than the more precise fasting levels. Last but not least, we have only included patients who were fit enough to receive TMZ-based chemoradiation, and this may have affected the survival outcomes. Nevertheless, we recruited a large patient cohort from 4 tertiary hospitals in Korea. To the best of our knowledge, this is the first study to reveal an association between the De Ritis ratio and survival outcomes in elderly patients with GBM.

In conclusion, our study showed that along with well-known prognostic factors such as KPS, MGMT status, and surgical extent, pre-RT serum biomarkers, including the De Ritis ratio and glucose level, also had prognostic value in elderly patients with glioblastoma treated with TMZ-based chemoradiation.

## Supplementary Material

vdad173_suppl_Supplementary_Figure

vdad173_suppl_Supplementary_Table

vdad173_suppl_Supplementary_Data
