# Supplementary material for: De Ritis ratio in elderly glioblastoma patients treated with chemoradiation: A comprehensive analysis of serum biomarkers
Source: Neurooncol Adv. 2023 Dec 28;6(1):vdad173. doi: 10.1093/noajnl/vdad173 (PMC10824161; doi:10.1093/noajnl/vdad173)
Supplement: vdad173_suppl_Supplementary_Figure [file vdad173_suppl_supplementary_figure.pptx]

## Slide 1
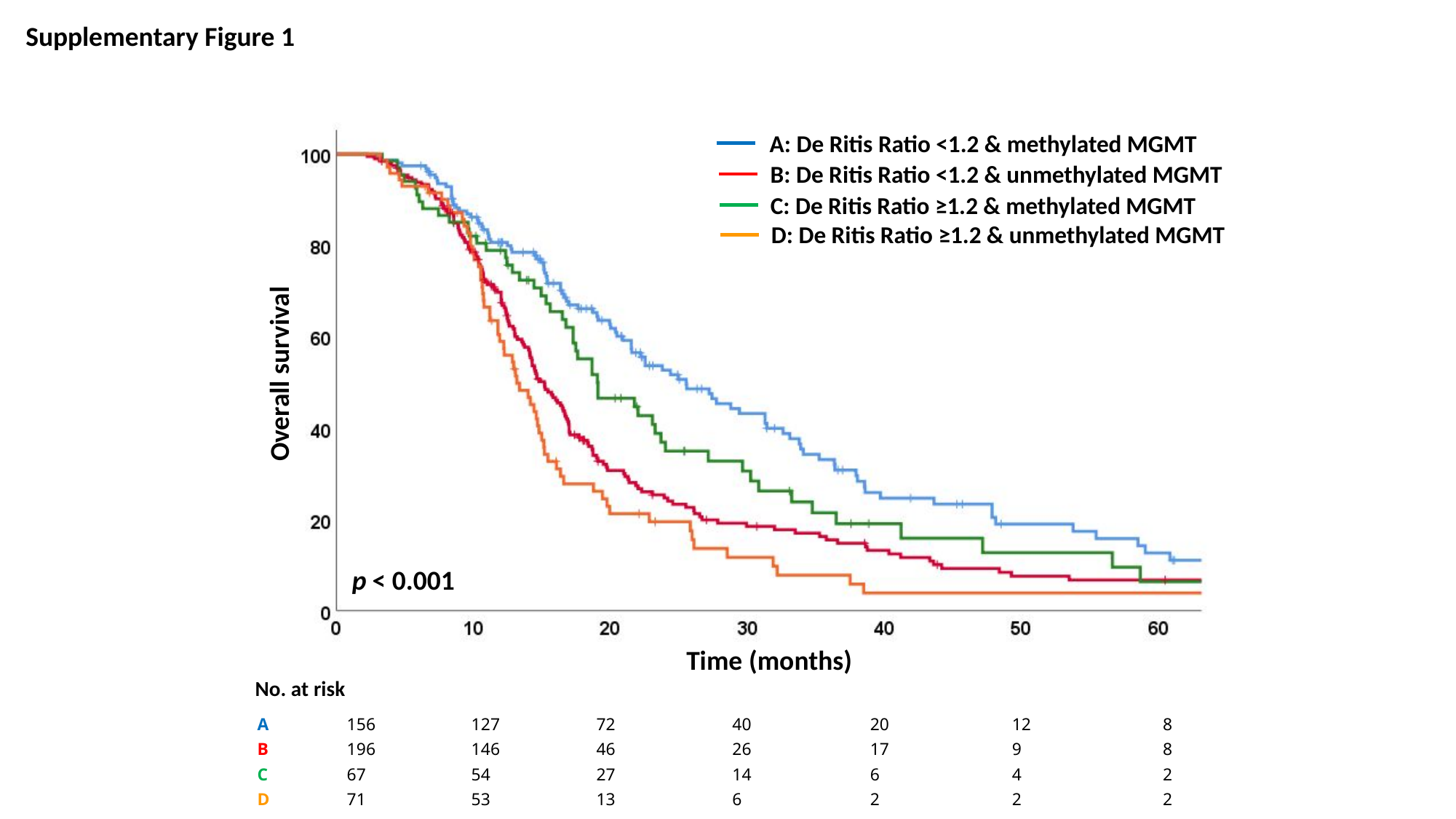

Supplementary Figure 1
A: De Ritis Ratio <1.2 & methylated MGMT
B: De Ritis Ratio <1.2 & unmethylated MGMT
C: De Ritis Ratio ≥1.2 & methylated MGMT
D: De Ritis Ratio ≥1.2 & unmethylated MGMT
Overall survival
p < 0.001
Time (months)
No. at risk
| A | 156 | 127 | 72 | 40 | 20 | 12 | 8 |
| --- | --- | --- | --- | --- | --- | --- | --- |
| B | 196 | 146 | 46 | 26 | 17 | 9 | 8 |
| C | 67 | 54 | 27 | 14 | 6 | 4 | 2 |
| D | 71 | 53 | 13 | 6 | 2 | 2 | 2 |
