# Supplementary material for: De Ritis ratio in elderly glioblastoma patients treated with chemoradiation: A comprehensive analysis of serum biomarkers
Source: Neurooncol Adv. 2023 Dec 28;6(1):vdad173. doi: 10.1093/noajnl/vdad173 (PMC10824161; doi:10.1093/noajnl/vdad173)
Supplement: vdad173_suppl_Supplementary_Table [file vdad173_suppl_supplementary_table.docx]

Supplementary Table 1. Univariate and multivariate analysis for overall survival in all patients, incorporating De Ritis ratio and serum glucose level as a single variable

|  | Univariate analysis | | |  | Multivariate analysis | | |
| --- | --- | --- | --- | --- | --- | --- | --- |
|  | HR | 95% CI | p value |  | HR | 95% CI | p value |
| Age (≥70 years vs. <70 years) | 1.33 | 1.08-1.63 | 0.007 |  | N.S. |  |  |
| KPS (≥70 vs. <70) | 0.53 | 0.43-0.65 | <0.001 |  | 0.53 | 0.42-0.65 | <0.001 |
| MGMT (methylated vs. unmethylated) | 0.57 | 0.46-0.70 | <0.001 |  | 0.54 | 0.44-0.67 | <0.001 |
| Temporal muscle thickness (normal vs. narrow) | 0.59 | 0.44-0.80 | 0.001 |  | N.S. |  |  |
| Resection extent (GTR, NTR, STR vs. PR, biopsy) | 0.66 | 0.53-0.84 | 0.001 |  | 0.74 | 0.58-0.94 | 0.013 |
| Neutrophil-to-lymphocyte ratio (<4.0 vs. ≥4.0) | 0.97 | 0.78-1.20 | 0.743 |  | N.S. |  |  |
| Platelet count (≥230 K/uL vs. <230 K/uL) | 0.91 | 0.74-1.11 | 0.351 |  | N.S. |  |  |
| SII (<730 x1000 cells/uL vs. ≥730 x1000 cells/uL) | 0.91 | 0.74-1.12 | 0.374 |  | N.S. |  |  |
| Group (DRR high & Glucose high vs. others) | 1.51 | 1.14-2.01 | 0.004 |  | 1.51 | 1.13-2.01 | 0.005 |
| Abbreviations: HR, hazard ratio; CI, confidence interval; N.S., not significant; KPS, Karnofsky performance status; MGMT, O6-methylguanine-DNA-methyltransferase; GTR, gross total resection; NTR, near total resection; STR, subtotal resection; PR, partial resection; SII, systemic immune-inflammation index; DRR, De Ritis ratio | | | | | | | |
|  |  |  |  |  |  |  |  |
|  |  |  |  |  |  |  |  |
|  |  |  |  |  |  |  |  |
