# Supplementary material for: De Ritis ratio in elderly glioblastoma patients treated with chemoradiation: A comprehensive analysis of serum biomarkers
Source: Neurooncol Adv. 2023 Dec 28;6(1):vdad173. doi: 10.1093/noajnl/vdad173 (PMC10824161; doi:10.1093/noajnl/vdad173)
Supplement: vdad173_suppl_Supplementary_Data [file vdad173_suppl_supplementary_data.docx]

**Supplementary Figure 1.** Kaplan–Meier estimates of overall survival of patients with (a) De Ritis ratio <1.2 & methylated MGMT, (b) De Ritis ratio <1.2 & unmethylated MGMT, (c) De Ritis ratio ≥1.2 & methylated MGMT, and (d) De Ritis ratio ≥1.2 & unmethylated MGMT.
